# Salvage of limb salvage in oncological reconstructions of the lower limb with megaprosthesis: how much to push the boundaries?

**DOI:** 10.1007/s00402-021-04165-8

**Published:** 2021-09-13

**Authors:** Matteo Innocenti, Francesco Muratori, Lorenzo Foschi, Saverio Bartolini, Maurizio Scorianz, Guido Scoccianti, Domenico Andrea Campanacci

**Affiliations:** 1 Department of Orthopaedic Surgery, Azienda Ospedaliero-Universitaria Careggi, Florence, Italy; 2 Department of Orthopaedic Oncology and Reconstructive Surgery, Azienda Ospedaliero-Universitaria Careggi, Largo Palagi 1, 50139 Florence, Italy

**Keywords:** Megaprostheses, Limb salvage, Oncological reconstructions, Revision surgery

## Abstract

**Introduction:**

Megaprosthesis represent the most commonly used limb salvage method after musculoskeletal tumor resections. Nevertheless, they are burdened by high complication rate, requiring several surgical revisions and eventually limb amputation. The aims of this study were to evaluate the effect of rescuing the limb with subsequent revisions on complication rates (a), incidence of amputations (b), and whether complications reduce functional outcome after the first surgical revision (c).

**Materials and methods:**

We retrospectively reviewed 444 lower limb megaprosthesis implanted for primary musculoskeletal tumors or metastatic lesions, from February 2000 to November 2017. 59 patients received at least one revision megaprosthesis surgery. MSTS score was used to assess final functional results. Complication-revision-amputation free survival rates were calculated both at 5 and 10 years of follow-up.

**Results:**

Complication free survival, revision free survival and amputation free survival at 10 years were 47% and 53%, 61% and 67%, 90% and 86% among all 444 patients and the group of 59 revised patients, respectively. The incidence of further complications after the first complication was 26% in the group treated with no subsequent revision surgeries and 51% in the group with at least one revision surgery. We found a trend of inverse linear relationship between the number of complications needing subsequent revision surgeries and the final MSTS.

**Conclusion:**

The number of further revision surgeries after limb salvage with megaprosthesis increases the incidence of complications. Repeated surgical revisions, in particular after infection, increase the amputation rate. The most frequent causes of failure were structural failures and infections. MSTS score was superior for patients undergoing limb salvage than amputees. However, MSTS progressively decreased with multiple revisions becoming inferior to the functional score of an amputated patient.

**Supplementary Information:**

The online version contains supplementary material available at 10.1007/s00402-021-04165-8.

## Introduction

Thanks to the advances in neoadjuvant therapies and diagnostic imaging, limb salvage surgery became popular in the treatment of extremities’ sarcomas, to the point of being carried out in more than 90% of cases [1].

Megaprostheses is the most frequently used method for limb reconstruction after resection of a musculoskeletal tumor of the extremities due to their availability, relative ease of use, immediate fixation, early weight bearing and rapid restoration of function [2–4]. Moreover, megaprosthesis are not subjected to allograft-related complications, such as fracture, non-union, and articular cartilage degeneration [5]. In particular, in patients who have already reached skeletal maturity, it is now the treatment of choice for osteoarticular reconstructions, preferable to the use of grafts [6]. However, despite all the improvements in surgical techniques and implant-related technologies, these implants are burdened by several complications. According to Henderson’s classification those are: soft-tissue failure, aseptic loosening, structural failure, infection, and tumor recurrence [3, 7–13]. Treatment of these complications can be devasting, due to the need of subsequential revision procedures that can eventually lead to an amputation [5].

The purpose of our study was to answer the following questions: (a) is the salvage of limb-salvage surgery with megaprosthesis, after a first revision prostheses surgery, burdened by a higher number of complications than primary implants? (b) Does the incidence of amputation increase with the number of subsequent revisions? (c) Do complications reduce functional outcome after the first surgical revision?

## Materials and methods

### Patient selection and clinical assessment

Authors implanted 444 megaprosthesis as a salvage surgical strategy of the lower limb at a single Institution from February 2000 to November 2017: 242 (54.5%) due to primary musculoskeletal tumors and 202 (45.5%) for metastatic lesions.

Among this series, we focused on patients who had undergone at least one revision surgery (group A). All the other patients with at least one complication but treated with a straightforward amputation or without substitution of any of the prosthesis’ components (group B) or patients who had not developed any post-operative complication (group C) were selected just as control group to run comparative analyses.

The group with at least one revision surgery (group A) included a cohort of 59 patients, 38 males and 21 females with a mean age of 43.5 years (range 12–87). Twenty-three had a right-sided tumor and 36 a left-sided one. The oncological diagnoses were as follow: osteosarcoma in 23 cases, ten metastatic diseases, nine chondrosarcomas, five Ewing sarcomas, four soft tissue sarcomas, three leiomyosarcomas, two giant-cell tumors of the bone, a giant-cell tumor of the tendon sheaths, a single multiple myeloma, and a Gorham disease.

Implant complications and failures were classified according to Henderson criteria [6].

Final functional results were assessed using MSTS score [13]. This score is calculated on the basis of a standardized physical examination with six criteria and differs slightly between the upper and lower limb. For the lower limb, the analysed factors are pain, function, emotional acceptance of the treatment outcome, need for walking aids, walking and gait.

### Implants and surgical procedure

Among the group A, we implanted 11 proximal femur megaprosthesis, 38 distal femur, four distal femur and proximal tibia megaprosthesis after extraarticular resection, five isolated proximal tibia, and one knee resection arthrodesis using a knee fusion megaprosthesis (in all cases we used the LINK® MEGASYSTEM-C®, Waldemar Link GmbH, Hamburg Germany) (Prosthesis details are reported in Table 1).

**Table 1:** Types of Megaprosthesis used

	No. of cases	Prosthesis type^a^	Cemented	Non-Cemented
Proximal Femur	11	Proximal Femur Megasystem-C + : 1 = Endoprostheses (Biarticular hip) 8 = Conventional THA (One Cup) 1 = Dual mobility THA (Bi-Mobile Cup) 1 = Antiprotrusio cage THA (PPR)	10	1
Distal Femur	38	Distal Femur Megasystem-C^b^	13 + 2 hybrid (1 cemented tibia only, 1 cemented femur only)	23
Knee Extrarticular Resection	4	Distal Femur + Proximal Tibia Magasystem-C^b^	2	2
Proximal tibia	5	Proximal Tibia Magasystem-C^b^	2	3
Knee arthrodesis	1	Knee Arthodesis Megasystem-C	1	0

^a^LINK® MEGASYSTEM-C®, Waldemar Link GmbH, Hamburg Germany

^b^Endo-Model SL Rotational Knee is used in the MEGASYSTEM-C

### Statistical analyses

Statistical analysis was performed using SPSS® statistics software (IBM®, Armonk, New York, USA). Simple descriptive statistics was used to calculate both the average, range, SD of MSTS scores, and to establish the percentages and incidences of complications and failures. The Student *t*-test was used to compare clinical outcome between patients with no megaprosthesis revision and those who had at least one revision surgery, taking a *p* values < 0.05 to be statistically significant. Kaplan–Meier analyses was performed to delineate complication free survival, revision free survival, amputation free survival rates both at 5 and 10 years of follow-up.

## Results

Among the whole series of 444 megaprosthesis, 332 patients had no complications (group C). 112 patients sustained at least one Henderson complication: 59 complications required at least one revision surgery (group A), 43 did not require any revision prosthetic surgery and ten patients were treated with a straightforward amputation and no implant revision surgery (group B = 43 + 10 patients).

The average follow-up of all 444 patients was 42 months (range 24–224); among 112 patients with one complication was 67 months (range 24–224); the average follow-up of the 59 patients with at least one revision surgery was 85 months (range 24–204).

Final oncological results of the cohort of 59 patients (group A) were no evidence of disease (NED) in 34 cases, alive with disease (AWD) in ten, dead of disease (DOD) in 14 and dead of other cause (DOC) in one case. The mean length of resection was 16.1 cm (range 7–30) in proximal femur, 17.2 (range 7–31) in distal femur and 11.2 cm (range 10–23) in the tibia. Mean surgical time was 213 min (range 115–300).

### Complications leading to the first and subsequent revision surgeries

**Fig. 1:**
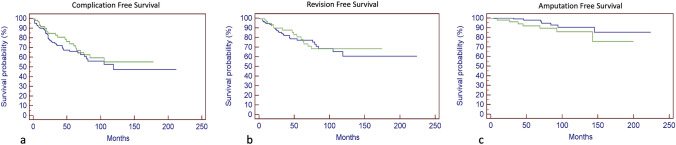
Kaplan Meir curves show complication free survival (**a**), revision free survival (**b**), and amputation free survival (**c**) of the whole population of 444 patients (blue lines) and of the 59 patients group A—green lines) who undergone at least 1 revision megaprosthesis surgery

Complications which have resulted in revision and subsequent re-revision surgeries are shown in Tables 2, 3, 4, 5 and 6. Surgical details of complications’ managements are described in “supplementary material”. Among 444 patients, 112 (25.2%) sustained at least one complication: 52.7% (59 patients, group A) of those patients underwent at least one surgical revision of the prosthetic components, while the other 47.3% (53 patients, group B) have been treated either non-surgically (31.1%) or surgically (46.6%) or with a straightforward amputation (22.2%). Group B sustained a subsequential 12 complications treated non-surgically. The incidence of new complications after the first complication was 51% in the group A and 26% in the group B. The complication free survival of the whole population of 444 patients was 66% at 5 years and 47% at 10 years, while the revision free survival was 77% at 5 years and 61% at 10 years, with an amputation free survival of 98% at 5 years and 90% at 10 years (Fig. 1a–c—blue lines).

**Table 2:** Complications leading to the first revision megaprosthesis surgery

Henderson type of complication	Proximal Femur	Distal Femur	Extraarticular Knee Resection	Knee arthrodesis	Proximal Tibia	Total
1A	4	/	/	/	1	5
1B	/	/	/	/	/	/
2A	1	5	/	/	/	6
2B	1	2	/	/	/	3
3A	1	16	2	/	/	19
3B	/	1	/	1	/	2
4A	1	12	1	/	2	16
4B	1	1	/	/	1	3
5A	/	/	1	/	1	2
5B	2	1	/	/	/	3
Total	11	38	4	1	5	59

**Table 3:** Complications leading to the second revision megaprosthesis surgery

Henderson type of complication	Proximal Femur	Distal Femur	Extraarticular Knee Resection	Knee arthrodesis	Proximal Tibia	Total
1A	/	/	/	/	1	/
1B	/	/	/	/	/	/
2A	/	/	/	/	/	/
2B	/	/	/	/	/	/
3A	/	4	2	/	1	7
3B	/	/	/	1	/	1
4A	/	5	/	/	1	6
4B	/	/	/	/	/	/
5A	/	/	/	/	/	/
5B	/	/	/	/	/	/
Total	/	9	2	1	3	15

**Table 4:** Complications leading to the third revision megaprosthesis surgery

Henderson type of complication	Proximal Femur	Distal Femur	Extraarticular Knee Resection	Knee arthrodesis	Proximal Tibia	Total
1A	/	/	/	/	/	/
1B	/	/	/	/	/	/
2A	/	/	/	/	/	/
2B	/	/	/	/	/	/
3A	/	2	/	/	1	3
3B	/	/	/	/	/	/
4A	/	1	/	/	/	1
4B	/	1	1	/	/	2
5A	/	/	/	/	/	/
5B	/	/	/	/	/	/
Total	/	4	1	/	1	6

**Table 5:** Complications leading to the fourth revision megaprosthesis surgery

Henderson type of complication	Proximal Femur	Distal Femur	Extraarticular Knee Resection	Knee arthrodesis	Proximal Tibia	Total
1A	/	/	/	/	/	/
1B	/	/	/	/	/	/
2A	/	/	/	/	/	/
2B	/	/	/	/	/	/
3A	/	/	1	/	1	2
3B	/	2	/	/	/	2
4A	/	1	/	/	/	1
4B	/	/	/	/	/	/
5A	/	/	/	/	/	/
5B	/	/	/	/	/	/
Total	/	3	1	/	1	5

**Table 6:** Complications leading to the fifth revision megaprosthesis surgery

Henderson type of complication	Proximal Femur	Distal Femur	Extraarticular Knee Resection	Knee arthrodesis	Proximal Tibia	Total
1A	/	/	/	/	/	/
1B	/	/	/	/	/	/
2A	/	/	/	/	/	/
2B	/	/	/	/	/	/
3A	/	/	1	/	/	1
3B	/	/	/	/	/	/
4A	/	1	/	/	/	1
4B	/	/	/	/	/	/
5A	/	/	/	/	/	/
5B	/	/	/	/	/	/
Total	/	1	1	/	/	2

Focusing on the group A of 59 patients, we reported the following primary complications requiring the first revision surgery: 5 type 1A, 6 type 2A, 3 type 2B, 19 type 3A, 2 type 3B, 16 type 4A, 3 type 4B, 2 type 5A, 3 type 5B (Table 2). The complication free survival was 71% and 53% at 5 and 10 years, respectively, with a revision free survival of 77% at 5 years and 67% at 10 years (Fig. 1a–c—green lines).

Among group A, complications requiring a second revision surgery (15 cases) are reported in Table 3. Nine of those were about distal femur megaprosthesis, two in the group of extraarticular resections, one in the knee arthrodesis prosthesis, and three in the proximal tibial megaprosthesis.

A total of six complications required the third revision: three for infection (4A in one and 4B in two cases) and three for breakage of prosthetic components (all of them was 3A) (Table 4).

The fourth revision was needed for five patients: three complications were about the distal femur megaprosthesis, and one in both the extraarticular knee resection and proximal megaprosthesis (Table 5).

Two patients also underwent a fifth revision, one for 3A and one for 4A complication (Table 6).

Finally, one (50% on two) patient underwent a sixth revision surgery, for 3A failure, and another one (50% on two) after five surgical revision was amputated for 4A complication. The patient who underwent a sixth revision was an extraarticular knee resection and was treated with a full implant revision, with the insertion of a new knee arthrodesis prosthesis.

Of the 59 patients of group A, seven were subsequently amputated (amputation’s incidence of 11.9%): four due to local recurrence (5A in two and 5B in two cases), two for infection (4A) and one for his own will for pain. The amputation free survival was 92% at 5 years and 85% at 10 years (Fig. 1c).

### Final functional results—MSTS score

**Fig. 2:**
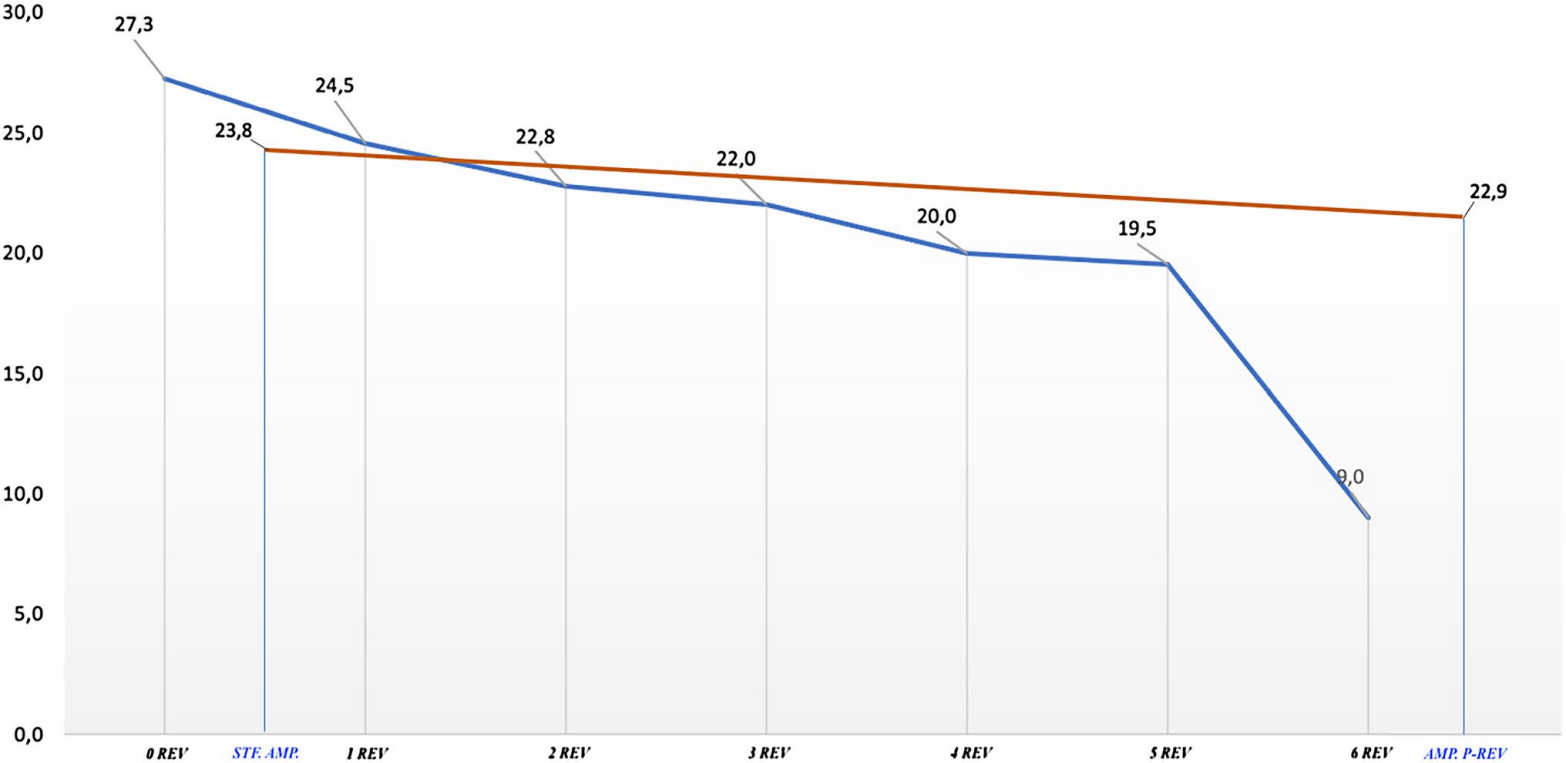
Final Functional MSTS scores of the whole study population of 444 patients. Blue line: MSTS scores of patients who has been treated with revision surgery progressively from zero up to six revision surgeries. Orange line: MSTS scores of patients who were treated with a straightforward amputation (*STF.AMP.*) and patients who got an amputation as the treatment of subsequent revision megaprosthesis’ complications (*AMP.P-REV*)

The final average MSTS score along with SD and score ranges of the entire study population are shown in Table 7. We found a trend of inverse linear relationship between the number of complications needing subsequent revision surgeries and the final MSTS: the higher the number of re-revisions per patient the worse the final MSTS (Fig. 2). The difference between the final MSTS score of patients who underwent zero revision surgeries and those who had at least one revision megaprosthesis surgery (*t* = 5.035; *p* < 0.001; Standard Error Difference = 0.923) was statistically significant in favour of the former group.

**Table 7:** MSTS score results

	No revision	Straightforward amputation	1 Revision	2 Revisions	3 Revisions	4 Revisions	5 Revisions	6 Revisions	Amputation after revision
Average	27.3	23.8	24.5	22.8	22.0	20.0	19.5	9.0	22.9
Range	9–30	18–28	13–30	5–28	21–23	19–21	19–20	9	20–28
SD	3.2	3.3	5.0	7.0	1.0	1.0	0.7	nd	2.8

## Discussion

The first purpose of our study was to analyse whether, after subsequent revision surgeries, the attempt to rescue the limb-salvage prosthesis surgery was subjected to an increase in the incidence of complications. 25.2% of patients of the whole study population had at least one complication, of which about half of those patients underwent a revision prosthesis surgery. The incidence of subsequent complications after the first complication was much higher in the group A than in the group that did not receive any revision prosthesis surgery (group B) (51% vs 26%). Therefore, the number of subsequent failures and megaprosthesis’ revisions seem to increase the risk of further of complications.

Analysing the site of failure, we observed that complication leading to revision of the megaprosthesis involved the distal femur or proximal tibia in 42.8% and the proximal femur in 10% of cases, respectively. Infection was the cause of failure in 33% of cases after the first revision, 40% of the second revision, 50% of third revision, and 20%, 50%, and 50% of the 4th, 5th, and 6th revision, respectively. Distal femur and proximal tibia site seemed to be a negative prognostic factor for infective failures.

Smolle et al. [14] reported a 65.4% of infective failures in the distal femur and proximal tibia versus 29% occurred in the proximal femur. Other authors reported similar data, albeit with lower incidence, confirming infection as the most frequent complication in megaprosthesis surgery [2, 3, 15, 16]. Our data confirm that the risk of infection in a patient with megaprosthesis after tumor resection remains high throughout life, increasing with repeated surgical procedures.

Nevertheless, contrarily to what have been reported in the largest retrospective multicenter study in literature regarding the mode of failure of those massive resection endoprosthesis [9], but in line with the report of Grimer et al. [17], the most frequent failure mode of megaprosthesis observed in our series was structural failure (type 3) instead of infection. It occurred in 35% of cases (21/59) requiring the first revision with component replacement. Actually, 19 type 3A failures were related to breakage of prosthetic components, while only two type 3B failures were periprosthetic fractures. The distal femur was the most affected site of structural failures (25.4%). In addition, the incidence of structural failure showed an increasing trend during the subsequent revision surgeries, representing the cause of failure in 40–50% of cases of further revisions after the first one. Henderson et al., despite reporting lower structural failures values compared to our study, they still had 23% of all failures in the distal femur and 18.8% in the proximal tibia [9].

In addition to structural failure, the other frequent complication reported in literature was aseptic loosening. Unwin et al. report aseptic loosening percentages of 6.2% for the proximal femur, 32.6% for the distal femur and 42% for the proximal tibia [18]. We could not confirm this trend in our study population. We reported only six Type 2 failures (1.4% among all 444 patients, 10% among group A of 59 patients). Differently from those Authors, as we already published elsewhere [19], prosthetic failure occurred in all cases at the morse taper (Waldemar Link) joining the different moduli.

The second analysed study end-point was to evaluate whether the incidence of amputation increases with the number of subsequent revision megaprosthesis surgeries. We observed a total of 17 amputations (15.8%) on 112 patients who had at least one complication. Ten out of 17 amputations have been performed as the final treatment of the first complication without undergoing any revision prosthesis surgery. The other seven amputations were about patients who received at least one revision surgery (group A), with an amputation’s incidence of 11.9%. In particular, four of those seven cases (57%) were observed as the treatment of complications after the first revision surgery, while the other three (43%) have been performed after the fourth and fifth revision, with an amputation free survival at 5 years of 92% and 85% at 10 years. Focusing on the relationship between the number of patients subsequently revised and the number of subsequent amputations, we have observed an increase in the relative amputations’ percentage incidence: 6.8% of incidence of amputation after the first revision, 33% of incidence after the fourth revision and 50% after the fifth. Moreover, all the amputations performed after the first revision surgeries were due to recurrence of the oncological disease, while two of the other three amputations were due to deep infections related to the multiple subsequent revision surgeries. The last amputation was about the patient willing of amputation in a young man who could not tolerate a painful knee due to repetitive revisions. Similar data are also reported in the literature where persistent infections represent the most common cause of amputation (from 23.5 to 87%) [4].

Finally, our study focused on the relationship between complications and the final functional clinical outcome. There are several studies reporting functional results after mega prosthesis implantation. The Musculoskeletal Tumor Society system (MSTS) [13] score is the most widely used evaluation system for quantifying function. As it was fully expected, the difference between the final MSTS score of patients who underwent zero revision surgeries (group B) and those who had at least one revision megaprosthesis surgery (group A) was significantly in favour of the former group independently by the number/type of complications. Among the group A, we found a trend of inverse linear relationship between the number of complications needing subsequent revision surgeries and the final MSTS: the higher the number of re-revisions per patients the worse the final MSTS. Furthermore, patients who received a straightforward amputation had a final MSTS score almost equal to the ones who underwent a single megaprostheses revision surgery. Similarly, patients treated with an amputation after previous single or multiple revisions reported a final MSTS score higher than patients with ≥ 2 revision surgeries independently from the type of complication leadings to amputation (Fig. 2).

Functional results data from our study confirm that limb-salvage with megaprosthesis is a successful and satisfying treatment for patients with lower extremity sarcomas. Based on final MSTS score, limb sparing surgery is also a preferable option than amputation. By the way, all that glittering ain’t gold. Limb salvage surgeries are complex procedures eventually followed by numerous complications and failures.

Our study has some limitations. First, it is a retrospective study. Secondly, it is based on a heterogeneous sample comprising both patients with primitive bone tumors and with metastatic lesions. Moreover, it includes both young and elderly patients with different functional demands. Finally, we considered/included different anatomical sites of the lower limb, that are biomechanically different and with different anatomical problems, whose complications can understandably be of different types. However, it also allows to underline the differences between the different anatomical sites.

Building upon our study/experience, if there are no contraindications dictated by the staging of the tumor or by other patient’s comorbidities we suggest to initially attempt to perform a lower-limb salvage surgery with megaprosthesis. In case of subsequent complications, except for the recurrence of the disease we suggest giving another try rescuing the limb-sparing surgery. However, at the occurrence of any other eventual complication after the second revision surgery, we also suggest taking into consideration the amputation as a definitive treatment, before the lower limb become completely non-functional or before getting subsequent complications and revisions that often lead to an amputation. Even though it is often initially emotionally hard to accept by patients, this definitive surgical procedure gives patients back their quality of life through time without wasting time in an endless circle of rescuing.

## Conclusion

Our study showed that the number of failures and further revision surgeries after limb salvage with megaprosthesis increase the incidence of complications. Repeated surgical revisions, in particular after infectious problems, increase the amputation rate. The most frequent causes of failure were structural failures and infections. The functional results assessed with the MSTS score were decidedly superior for patients undergoing limb salvage than amputees. However, MSTS progressively decreased with multiple revisions becoming inferior to the functional score of an amputated patient.

## Supplementary Information

Below is the link to the electronic supplementary material.

Supplementary file1 (DOCX 15 kb)
