# Supplementary material for: Salvage of limb salvage in oncological reconstructions of the lower limb with megaprosthesis: how much to push the boundaries?
Source: Arch Orthop Trauma Surg. 2021 Sep 13;143(2):763–71. doi: 10.1007/s00402-021-04165-8 (PMC9925554; doi:10.1007/s00402-021-04165-8)
Supplement: Supplementary file 1 — Supplementary file1 (DOCX 15 kb) [file 402_2021_4165_MOESM1_ESM.docx]

*Complications requiring the first revision*

We reported the following primary complications: 5 type 1A, 6 type 2A, 3 type 2B, 19 type 3A, 2 type 3B, 16 type 4A, 3 type 4B, 2 type 5A, 3 type 5B (Table 2).

In the proximal femur megaprosthesis group we had 11 patients with at least one surgical complication that required a surgical revision: 4 were type 1A, all treated with a revision of the acetabular cup; 1 was 2A treated with a revision of the femoral stem; 1 was 2B treated with replacement of the implant with a composite prosthesis; 1 was 3A treated with a new megaprosthesis implant; 1 was 4A treated with a two stage revision and the implant of a silver coated megaprosthesis; 1 was 4B treated at another institution; 2 were 5B, one treated with a total femur resection and megaprosthesis, and one treated with hemipelvis resection, massive graft and new acetabular component.

In the distal femur group we had 38 patients with at least one complication that required a surgical revision: 5 were 2A, 4 treated with a revision of the femoral stem and one with implant of a total femur megaprosthesis; 2 were type 2B both treated with a revision of the femoral stem; 16 were 3A all treated with a revision of the femoral stem; 1 was 3B treated with osteosynthesis; 13 were 4A, 3 treated with a one-stage revision and joint components replacement, 8 with two-stage revision (2 with knee arthrodesis prosthesis, 2 with silver coated megaprosthesis, 1 with joint components revision, 3 with a new distal femur implant), one with a medial gastrocnemius rotation flap and patella cerclage, and the last one with the revision of the femoral component with a silver coated component leaving the stem in situ; 1 was type 4B treated with a one-stage revision; 1 was type 5B treated with excision of recurrence and implant of a new megaprosthesis.

In extraarticular knee resection cases we observed 4 patients with at least one complication that required a surgical revision: 2 were type 3A, one treated with revision of femoral stem and joint component, and the other one with revision of both femoral and tibial stem; 1 was 4A treated with a two-stage revision and implant of a knee arthrodesis nail; 1 was 5A treated with total resection of femur and total femur prosthesis.

In proximal tibia resection group we recorded 5 patients with at least one complication that required a surgical revision: 1 was type 1A treated with revision of femoral and joint component; 2 were 4A, one treated with two-stage revision and implant of knee arthrodesis prosthesis, and the second treated with a one-stage revision, implant of a knee megaprosthesis and a coverage rotational medial gastrocnemius flap; 1 type 4B treated with one-stage revision and implant of new tibial component; 1 was type 5A treated with excision of recurrence and new tibial component.

In the knee arthrodesis we had one type 3 B complication treated with revision of the coupling component.

*Complications requiring the second revision*

In proximal femurs we had no surgical complication after the first revision.

In distal femur we had 9 complications after the first revision that required a new surgical revision; 4 were type 3A, 3 treated with a revision of the femoral stem and one with a custom made megaprosthesis; 5 were 4A, 3 were treated with a two-stage revision, one with the removal of the plate, previously inserted for a periprosthetic fracture synthesis, and the last one was treated with joint component replacement. We also had 4 complications that undergo to amputation, 2 were 5A and 2 were 5B.

In the extraarticular knee resection we observed 2 complications after the first revision that required a new surgical approach, both were type 3A and were treated with the implant of a knee arthrodesis prosthesis.

In knee arthrodesis prosthesis we had 1 complication after the first revision, it was a 3B and treated with a new knee arthrodesis prosthesis.

In the proximal tibial megaprosthesis group we observed 3 complications after the first revision that required a new revision surgery, 1 was type 1A treated with revision of femoral component, 1 type 3A treated with a revision of joint component, 1 type 4A treated with two-stage approach and implant of a new tibial stem. (Table 3)

*Complications requiring the third revision*

Six (40% on 15) patients needed a third surgical revision, 3 for infection (4A in 1 and 4B in 2 cases) and 3 for breakage of prosthetic components (all of them was 3A) (Table 4).

In distal femur we had 4 complications after the second revision that required a third revision surgery; 2 were type 3A, one treated with a femoral stem revision and the other one with entire prosthesis revision; 1 was type 4A treated with a two-stage revision and implant of a knee arthrodesis prosthesis; 1 was 4B treated with two-stage revision and implant of a new megaprosthesis.

In extraarticular knee resection we had 1 patient who underwent a third revision surgery, due to 4B complication, treated with a two-stage revision and implant of a knee arthrodesis prosthesis.

In proximal tibia we had 1 patient who underwent a third revision surgery after a 3A complication, treated with revision of the modular component.

*Complications requiring the fourth revision*

Five (80% on 6) patients underwent a fourth revision, 2 for 3A, 2 for 3B and 1 for 4A failure (Table 5).

In distal femur we had 3 complications who required a fourth revision surgery; 2 were 3B, one treated with osteosynthesis, and one treated with the implant of a new knee arthrodesis prosthesis; 1 type 4A, treated with a two-stage revision and implant of knee arthrodesis prosthesis.

In extraarticular knee resection we had 1 patient who underwent a fourth revision surgery for a 3A complication, treated with a revision of the joint component of the knee arthrodesis nail.

In proximal tibia we had 1 patient who underwent a fourth revision surgery after 3A complication, treated with a revision of the modular component.

*Complications requiring the fifth revision*

Two (40% on 5) patients also underwent a fifth revision, 1 for 3A and 1 for 4A complication.

In distal femur we had one patient who underwent a fifth revision surgery. It was due to a 4A complication, treated by surgical scar and bone debridement, washing but retention of the implant and augmentation with cement.

In extraarticular knee resection we had one patient who underwent a fifth revision surgery, it manifested a 3A complication, treated with middle prosthesis body revision.

*Complications requiring the sixth revision*

Finally, one (50% on 2) patient underwent a sixth revision, for 3A failure, and another one (50% on 2) after five surgical revision was amputated for 4A complication.

The patient who underwent a sixth revision was an extraarticular knee resection and was treated with a full implant revision, with the insertion of a new knee arthrodesis prosthesis.
